# “I am Primarily Paid for Publishing…”: The Narrative Framing of Societal Responsibilities in Academic Life Science Research

**DOI:** 10.1007/s11948-020-00191-8

**Published:** 2020-02-11

**Authors:** Lisa Sigl, Ulrike Felt, Maximilian Fochler

**Affiliations:** 1 Research Platform Responsible Research and Innovation in Academic Practice, University of Vienna, Universitätsstrasse 7, Vienna, 1010 Austria; 2 Department of Science and Technology Studies, University of Vienna, Universitätsstrasse 7, Vienna, 1010 Austria

**Keywords:** Responsible research and innovation, Narrative infrastructure, Societal relevance, Interaction, Diligence, Communication

## Abstract

Building on group discussions and interviews with life science researchers in Austria, this paper analyses the narratives that researchers use in describing what they feel responsible for, with a particular focus on how they perceive the societal responsibilities of their research. Our analysis shows that the core narratives used by the life scientists participating in this study continue to be informed by the linear model of innovation. This makes it challenging for more complex innovation models [such as responsible research and innovation (RRI)] to gain ground in how researchers make sense of and conduct their research. Furthermore, the paper shows that the life scientists were not easily able to imagine specific practices that would address broader societal concerns and thus found it hard to integrate the latter into their core responsibilities. Linked to this, researchers saw institutional reward structures (e.g. evaluations, contractual commitments) as strongly focused on scientific excellence (“I am primarily paid for publishing…”). Thus, they saw reward structures as competing with—rather than incentivising—broader notions of societal responsibility. This narrative framing of societal responsibilities is indicative of a structural marginalisation of responsibility practices and explains the claim, made by many researchers in our sample, that they cannot afford to spend time on such practices. The paper thus concludes that the core ideas of RRI stand in tension with predominant narrative and institutional infrastructures that researchers draw on to attribute meaning to their research practices. This suggests that scientific institutions (like universities, professional communities or funding institutions) still have a core role to play in providing new and context-specific narratives as well as new forms of valuing responsibility practices.

## Introduction

There is a long-standing debate about how far the engagement with the societal implications of emerging sciences and technologies has been able to change the cultures in which research is carried out. Since the early 1990s at the latest, the life sciences have been amongst the most heavily researched fields with regard to its societal implications. In the US and many European countries like Austria, the Netherlands or the United Kingdom, special research programmes for the study of ethical, legal and social implications or aspects (ELSI/ELSA, hereafter called ELSA) have sparked an impressive body of research on societal issues and concerns (see Hilgartner et al. 2017 for an overview). These studies had much success in drawing public attention to topics such as emerging biotechnologies and their potential implications for health, agriculture or industry. However, a number of researchers who participated in these studies began to question how successful these programmes were when it came to changing actual research practices. Viseu (2015), for example, noted with concern that too often, the study of ELSA failed to live up to its potential. Instead of being a joint effort between social science and humanities (SSH) scholars and researchers in the sciences, there was a regularly a kind of division of labour: SSH scholars reflected on the possibilities for change, but there were no incentives for life scientists to really engage in changing the way they framed and conducted their research (cf. Felt et al. 2013; Rabinow et al. 2012):

“[T]he other scientists seemed to view my role as one of managing a narrow list of possible risks and consequences, so that if a researcher followed my instructions and ticked boxes, then I would bless them as ‘social and ethical’ and they would be free to do their work with no concerns. I was routinely (wrongly) introduced as an ethicist and was expected to find minimal, non-disruptive ways of dealing with social and ethical issues.” (Viseu 2015)

The expectation that ELSA should be about outsourcing non-disruptive reflection to SSH scholars often rendered ELSA research a mere early warning of potentially negative implications, but it did not lead to any deeper questioning of the research process more generally. While such approaches were in many ways very successful at unpacking the societal implications and risks related to a research field, they were often unable to engender new research trajectories that actually responded to societal issues and concerns.

Criticism of this lacuna, amongst others, has triggered a shift in emphasis to more interactive forms of reflection (interdisciplinary engagement but also exchanges between scientists and other societal actors) in more recent attempts to anchor societal responsibilities in research and innovation practices. For example, responsible research and innovation (RRI) in the European framework programme, Horizon 2020, highlights the need for such closer articulation of scientific and societal dimensions. More so than the earlier ELSA-approaches, this call for interaction demands that different actors actually take on responsibility in their practices:

“Responsible research and innovation (RRI) implies that societal actors (researchers, citizens, policy makers, business, third sector organisations, etc.) work together during the whole research and innovation process in order to better align both the process and its outcomes with the values, needs and expectations of society.” (European Commission 2019)

When RRI as a concept started to take shape in the run-up to Horizon 2020, it seemed to represent “an emerging zeitgeist for ‘responsible innovation’ that may intuitively feel right” but which still exhibited “a lack of clarity in terms of definition [and] practice” (Owen et al. 2012). RRI was also heavily loaded with expectations: most importantly, that research would become “responsive” to societal issues and concerns, i.e. that research processes would be reshaped to trigger “innovation that looks different” (Owen et al. 2012). Crucially, this implies a second expectation, namely that researchers reframe and broaden what they see as their responsibilities, from traditional responsibilities, such as research integrity, to broader responsibilities of considering societal issues and concerns and responding to them in their research practices (Owen et al. 2012). For this reason, the term ‘responsibility’ was put centre stage to express the expectation that these interactions are meant to change both research processes and their outcomes.

These international developments around ethical, legal and social aspects in science have also been momentous for the Austrian context. Besides Austrian researchers’ participation in research projects of European framework programmes that have increasingly emphasised societal aspects (starting with the 5th framework programme in the late 1990s; Felt and Wynne 2007), other programmes have been set in place to foster what we today often include as RRI. Amongst the larger of these efforts to foster reflections in this regard were ELSA-projects in a large genome research programme (GEN-AU/Genome Research in Austria), a citizen science funding programme (Sparkling Science) and initiatives directly inspired by RRI (e.g. Initiative Responsible Science, Research Platform Responsible Research and Innovation in Academic Practice). These programmes have sparked debates within research communities about what it means to do socially responsible research. However, it is unclear to what degree these debates have actually resulted in sustainable shifts in how researchers integrate societal responsibilities into their self-understanding and into their practices of knowledge production (de Saille 2015).

To address how researchers think about societal responsibilities, and how far they relate these societal responsibilities to their work practices, the four central research questions (RQ) of this paper are: how researchers narratively frame societal responsibilities (RQ1), how they assume certain responsibilities in practice (RQ2), whether and how they ascribe (or delegate) certain societal responsibilities elsewhere (e.g. to other actors, institutional contexts or points in time) (RQ3), and how far this narrative framing relates to social and institutional contexts (RQ4). The study builds on 11 group discussions and 28 interviews (with a total of 112 participating researchers, amounting to 1033 pages of transcripts). The discussions and interviews build on a card-based methodological design (Felt et al. 2018b) that was specifically developed to facilitate discussions about societal responsibilities in life science research. The two main interests in this methodological approach are to understand (a) how researchers narratively frame their responsibilities and (b) whether and why they delegate some responsibilities elsewhere.

This paper builds on the findings of previous studies showing that, while researchers tend to see abstract concepts like RRI as irrelevant to them, they assumed a range of bottom-up responsibilities in their practices. Glerup and colleagues, for example, identified “producing sound science, taking care of employees, creating ‘impact’ and carrying out publicly legitimate science” to be a set of such responsibilities (Glerup et al. 2017). Such findings suggest that understanding which responsibilities researchers assume in practice (and why they do so) requires an in-depth analysis of how they narratively frame different kinds of responsibilities. This paper puts the analysis of such narratives centre stage. As we will demonstrate, this analytical focus on narratives allows us to grasp some of the more tacit aspects in research cultures that guide researchers’ reasoning and that allow them to weave aspects of societal responsibility into what they see as their core practices (or that prevent them from doing so). In this way, the paper contributes to an approach that takes researchers’ perspectives seriously in order to contribute to effective policies of increasing societal impact and the public value of science (cf. de Jong et al. 2016).

## Approaches to Anchoring Societal Responsibility in Scientific Cultures

One approach to anchoring societal responsibilities in research cultures is to adapt institutional framework conditions (e.g. in career systems or evaluation systems; Fochler et al. 2016). Research has been done in this regard with a predominant focus on reducing institutional barriers (e.g. EU projects like HEIRRI/Integrating RRI into Higher Education Institutions, NUCLEUS/Bringing RRI to Life in Universities and Scientific Institutions, MoRRI/Monitoring the Evolution and Benefits of RRI) and developing measures for making responsibility an institutionalised ambition (Felt 2017; Felt et al. 2018a; Lindner and Kuhlmann 2016). To complement this approach, research has aimed at providing individuals with tools and methods for how to put responsibility into practice (for examples, see the database of the RRI Tools project^1^). These approaches implicitly follow the liberalistic idea that, once institutional barriers are removed and researchers are provided with a repertoire of tools for practicing RRI, researchers will be free to practice societal responsibility.

As has been noted, however, the risk is high that such approaches are interpreted as a universalistic, one-size-fits-all approach to anchoring RRI in research practices (Groves 2017). This has given rise to two sets of concerns: The first is that, in these approaches, the concrete actors and research communities, with their social and epistemic specifics, tend to remain abstract and anonymous: Davies and Horst state that “it is striking that the individual is de-emphasised in this discussion” and that “the actors in RI [research and innovation] are construed in terms of large, general categories, with little reference to particular, specific individuals or indeed to individual action at all” (Davies and Horst 2015: 50). Although, in their perspective, RRI is a governance tool that should “turn away from top-down, command and control regulation and (move) towards a broader distribution of ‘soft law’ activities”, it often remains unclear how individual actors are meant to act in order to realise RRI (Davies and Horst 2015: 51).

Linked to this, the second concern is that universalistic approaches to RRI run the risk of missing context-specific conditions (e.g. disciplinary, cultural, institutional) and overlook the importance of dominant narrative infrastructures, i.e. the shared set of reference narrations that are temporally stable and available for sense-making within particular groups/environments, and which may facilitate or prevent societal responsibility from becoming a core academic value (Felt 2017). Only recently, Delgado and Åm emphasised that what societal responsibility can mean in concrete research settings must remain an empirical question. For indeterminate processes like research, the actors involved must find situated ways of reflecting on societal issues and concerns and staying responsive to the often unpredictable turns research may take (Delgado and Åm 2018).

These lines of reasoning strongly suggest that, to make a difference in actual research practices, approaches to RRI need to take into account the social and epistemic specifics of each concrete research setting. In other words: there should be space to build specific practices of (or tools to foster) societal responsibility from within research contexts (instead of importing generic approaches). This resonates with other voices who are concerned about a “fetishizing of” (Groves 2017) or tick-box mentality (Felt 2017) regarding certain responsibility practices (such as public engagement). These voices also argue that it is necessary to move beyond the aim of “standardising” the meaning of responsibility, and allow for context-specific diversity in meanings and practices of responsibility (e.g. Wickson and Forsberg 2015). Such arguments build on recent studies that have demonstrated the importance of analysing context-specific and often tacit (e)valuation processes. Doing so allows us to grasp how researchers are socialised into context-specific ways of perceiving different kinds of responsibilities and values as relevant to them and of prioritising responsibilities in their actual practices. Fochler and colleagues have, for example, shown how PhD students at the beginning of their careers “relate to a wider evaluative repertoire” when it comes to assessing what they should and want to research, whereas postdocs seem less able to see solving societal problems and meeting societal needs as a priority in their research work. The authors conclude that “academic socialization in the life sciences seems to structurally inhibit engaging with societal responsibility rather than fostering it” (Fochler et al. 2016). This suggests that there is a strong link between the specific (e)valuation regimes in research cultures and the ways in which researchers value and plan their research practices and learn to assume and/or ascribe responsibilities in their actual practices (cf. Müller and de Rijcke 2017).

The challenge of putting RRI into practice thus lies in understanding how far (and which) ideas of societal responsibility can gain legitimacy in situated cultures of knowledge production and their systems of valuation and reward. This paper takes the above concerns seriously and provides an empirical analysis of the perspectives of life scientists on whether and how they want to assume societal responsibilities, and how they position themselves in relation to different kinds of societal responsibilities. We can understand this positioning also in terms of sketching a “geography of responsibilities” (Felt 2017). Using the metaphor of a geography helps us to imagine how researchers position themselves within an imagined map of responsibilities, where some responsibilities appear close enough to be considered in their research practices, while other responsibilities are imagined as being too far away to matter in researchers’ situated research practices.

## Conceptual Approach: Lived Narratives and Narrative Infrastructures

To understand how researchers frame their societal responsibilities (i.e. how they sketch their geography of responsibilities), we analyse the core narratives that guide them in talking about assuming and ascribing responsibility. Narratives in this context are understood as culturally anchored stories that give meaning and legitimacy to practices. Analysing narratives thus makes it possible to understand how researchers make sense of societal responsibilities in life science research cultures, but also to see what responsibilities are ascribed elsewhere (to other actors, institutions, places, times) and what kinds of practices count as legitimate practice in life science research.

This conceptual approach builds on the core assumption that practices of scientific knowledge production are tightly entangled with broader societal processes of meaning-making, and with normative assumptions about what is worth being considered in research and what is not. According to Sheila Jasanoff, an important moment in this articulation—or “co-production”—of science and society are discourses (or “narratives”): actors can shape this co-production, for example, by appropriating “existing discourses” and “selective retailoring to suit new needs” (2004: 41). Researching narratives thus makes it possible to understand important characteristics of the co-production of research and society, and how it is stabilised in specific current research environments.

This conceptual approach assumes that, despite situated differences in how life scientists assume and ascribe societal responsibilities, they share *a set of narratives* that allow them to make sense of this positioning work. While researchers use these narratives in diverse ways to sketch their individual geography of responsibilities and position themselves within it, in doing so they share a set of narrative reference points (a narrative infrastructure). This allows researchers to individually and collectively make sense of their research practices in their particular environments, but in turn it also guides how researchers think about and carry out their research. The spatial metaphors our conceptualisation uses (geographies, infrastructures) imply that researchers can move within these narratives and shape them while doing so.

Particularly for knowledge-based work environments like research, it has been emphasised that narratives guide the ways in which individuals learn to give meaning to how they operate in communities of practice (cf. Akrich 2010). Narratives facilitate sense-making practices, particularly in conditions of complexity and unpredictability, and serve as “modes of knowing” that carry information about what counts as accepted practice (Czarniawska-Joerges 2004). While narratives guide practices and are “constitutive of agency” (cf. Deuten and Rip 2000), researchers also develop meanings out of their practical experiences, which may then be “narrativised” and can thus renew these guiding practices (Boland and Tenkasi 1995: 357).

In this sense, narratives allow scientific communities to communicate shared values and make sense of practices. They can be understood as stabilising elements in that they help to define and reproduce research cultures. Analysing narratives thus makes it possible to understand how certain ideas are stabilised (or marginalised) within particular communities. This is because “narratives can take different forms, including assessments, reconfigurations of past developments, future-oriented accounts voicing promises […] but also potential threats and moral reflections of what is good science and innovation and how a good researcher should be” (Felt 2017). At the same time, however, there is also variation in how people “live” these narratives such that they change them or their meaning (cf. van der Burg 2016). Thus, on the one hand, analysing narratives helps us to understand how geographies of responsibility are stabilised, and which actors are seen to be responsible to society, in what ways, and through which practices. On the other hand, by investigating the diverse ways that narratives are used to express the expectations and practices of individuals (cf. Larkin 2013), we may also learn about potential trajectories of change in research cultures. As Ulrike Felt put it, studying narratives is vital for “understand(ing) change beyond formal structural shifts.” (Felt 2017: 55).

Together, a set of evolving narratives in a specific context builds a narrative infrastructure “that enables and constrains the possible stories, actions and interactions by actors” (Deuten and Rip 2000). In asking how researchers sketch their geography of responsibility, we understand researchers’ narrative infrastructure to be a language “through which meanings and values of academic knowledge/work and its relation to society can be articulated, circulated and exchanged across space and time” (Felt 2017). It serves as a frame of reference that allows researchers to make sense of their research processes (Felt 2017) and enables the communication of and reflection on complex and rather unfamiliar issues such as, in our case, the responsibilities between science and society.

Analysing narrative infrastructures and how they are used allows us to understand how far societal responsibilities have become part of researchers’ *epistemic living spaces*, “the multi-dimensional structures—symbolic, social, intellectual, temporal and material—which mould, guide and delimit in more or less subtle ways researchers’ (inter)actions, [and] what they aim to know” (Felt 2009). This serves as a basis to discuss how far material systems, administrative techniques and political rationalities stabilise certain notions of societal responsibility in research, while potentially marginalising others.

## Methods

A core aim of the study is to understand how researchers think about societal responsibilities, and how far they relate these societal responsibilities to their everyday work practices. To grasp this, we needed to understand how researchers make sense of their research practices, and how they narratively frame societal responsibilities in relation to their research within their specific social and institutional contexts.

The challenge in studying narrative infrastructures lies in encouraging researchers to bring to the surface tacit considerations that they are often not used to discussing on an everyday basis and that many of them have not yet consciously reflected upon. To facilitate this, it was necessary to develop a methodological approach that allowed researchers to make explicit the implicit ideas and narratives they are motivated by and that guide their practices. Methods of qualitative social science research are uniquely suited for this task for several reasons. On the one hand, qualitative methods allow us to study not only which values researchers hold and which practices they engage in, but how they narratively make sense of these values and practices and relate them to each other. On the other hand, qualitative methods allow for more interaction between the participants of a study (e.g. interviewees) and those conducting a study, which facilitates in-depth narrative reflection (Silverman 2015).

This study builds on extensive empirical material consisting of group discussions and qualitative interviews with life scientists in Austria between 2016 and 2018 that were all conducted by the authors of this paper. In total, 112 researchers have participated in this study, 97 of whom participated in 11 group discussions (à 3 h). In the group discussions, 6 PIs, 30 postdocs, 49 PhDs, 8 master’s students and 4 lab managers participated. 6 of the group discussions included all career stages (usually 1 PI, 1–2 postdocs, 3–4 PhD and master’s students), 2 group discussions were conducted with postdoctoral researchers and 3 group discussions were conducted with PhD students. Furthermore, we conducted 28 interviews (à app. 1 ½h): 13 interviews with PhDs who had participated in group discussions earlier and 15 additional interviews with 15 PIs (who had not participated in group discussions). Overall, 1033 pages of transcripts were analysed for this study.

The group discussions and interviews took place at six research institutions that all play central roles in the life science research community in Vienna, not least because three of four of these institutions run a joint international PhD school with considerable influence in the field. Participants were recruited via previous cooperation partners, leading researchers at the respective research institutions, the coordinators of PhD schools, and via individual e-mail invitations.

Both group discussions and interviews built on the card-based discussion method called “IMAGINE RRI” (Felt et al. 2018b). The method was specifically developed to trigger reflection about what constitutes a responsible researcher and what responsible research can look like in practice. The group setting takes roughly 3 h and has three successive discussion steps. For each step, researchers were provided with cards representing different repertoires of statements and discussion questions to provoke a debate and enable them to formulate their own position. In each step, every researcher got the opportunity to formulate his/her own statement, as well as to comment on and discuss the statements made by the other discussants.

In a first step, 12 “statement cards” were provided to support their reflection process.^2^ The statements on the cards reflected a broad repertoire of ideas, some representing principles of RRI (such as that science should interact with other societal actors) and some representing ideas that advocate for clear boundaries between science and society. Researchers were asked to pick one card that they agree with and one card that they disagree with and then explain their choices individually before discussing their position in the group. This first step of the discussion deliberately offered researchers publicly available narratives and encouraged them to explain their own positions in relation to these narratives. In case they wanted to formulate and add additional narratives, empty cards were provided, but were rarely used. Instead, researchers’ own statements were inspired by the text on the cards but were usually creatively rearranged and supplemented to form new individual statements. Frequently, researchers also used the cards flexibly—e.g. by picking more cards than they were asked or by citing shorter fractions of statements to explain their own positions, assembling step by step their very own narratives of making sense of their societal responsibilities.

In the next discussion step, 15 “practice cards” were provided, each with a cartoon and two controversial questions that address specific practices and decisions in the life sciences where questions of responsibility play a role (e.g. choosing research questions or communicating with societal actors). Each participant again chose two cards and explained how relevant they are to their own practices.

The third discussion step addressed the role of institutional contexts for researcher’s ability to act upon their responsibilities. In this step, 13 “context cards” provided narrations about how far institutional contexts of research (e.g. competition, evaluations, career structures) support or obstruct responsible practices.

The 28 interviews also followed this card-based approach in order to facilitate a conversation between interviewer and interviewee on societal responsibilities in research.

In both group discussions and interviews, the card-based approach had the particular advantage of organising the group discussions and interviews along three steps (statements, practices, contexts), which allowed researchers to reflect on societal responsibilities from different perspectives (including their own implicit assumptions, culturally anchored practices and institutional conditions) and to make explicit their narrative infrastructure within a relatively short time frame.

The analysis of group discussions and interviews by the authors of this paper was guided by a grounded theory approach (Charmaz 2006; Glaser and Strauss [1998]2005). The analysis started with coding the transcript material (assisted by the software Atlas.ti) and discussions amongst the authors about what the main themes across the material were that are relevant with regard to the study’s research questions. Hypotheses developed in this initial coding were then refined using the logic and techniques of abductive analysis (Timmermans and Tavory 2012). Given the high number of participants and interviews/discussions, Atlas.ti was also helpful in identifying the density and frequency of specific narratives.

## How Life Science Researchers Conceptualise Their Responsibilities to Society

The following section reports the main narrative lines along which the participants in our card-based discussions and interviews situated themselves in relation to different kinds of societal responsibilities. The quotes we use in this section are exemplary, i.e. they can be read as representative for a larger set of similar statements made by researchers in our sample.

The first part (‘Three main narratives about being a responsible researcher’) is structured around three main narratives—being a diligent researcher, communicating with society and articulating societal relevance—that serve as reference points in talking about responsibility in relation to researchers’ practices (i.e. that make up a narrative infrastructure) (RQ1). While examining these three narratives, we attend to how researchers assume certain responsibilities in practice (RQ2) and what kinds of societal responsibilities they assume themselves or ascribe elsewhere (RQ3). In the second part (‘Two narratives that stabilise the broader narrative infrastructure for being a responsible researcher’), the paper traces two important characteristics of this narrative infrastructure that relate to social and institutional contexts of research (RQ4). As we discuss below, these contexts help to explain why the narrative infrastructure emerging from our data is relatively stable and seems to remain rather undisturbed by policy initiatives like RRI.

### Three Main Narratives About Being a Responsible Researcher

#### Being a Diligent Researcher

The most dominant and least controversial narrative researchers use in talking about societal responsibility was related to good inner-scientific practices. Many discussants picked and agreed with a card that framed research integrity, in the sense of diligently conducting experimental practice, as the most important element in being a responsible researcher. Consider how a PhD student explains this:

PhD2^3^: [A] favourite of all of us is the diligence card… I feel like this is the very first step in the way to… be a responsible researcher. … if you conduct your research in a proper way then this entails everything else. So, if you record your results properly then… the funding that will be given based on these records and these data … to things that are also worth investigating. And if people commit fraud at this very, very basic level of research then this basically destroys the whole ecosystem of research … this is really the base responsibility of any … person in this kind of profession.

As in this quote, researchers saw maintaining responsible conduct as the mandatory responsibility of every individual scientist. By choosing this card, the discussants assumed that science’s most important responsibility to society was to produce reliable knowledge. Research misconduct is then seen as the main threat to this practice:

PD2: Sort of the primary dogma, that we should all, let’s say, work on is that, you know, a responsible researcher really conducts his or her research to… the best of their ability and … also sort of diligently so that it’s controlled and it’s … reproducible … With the best possible models or systems … this is sort of … the basis should be this.

Besides being considered a responsibility towards society, diligence was also seen as a responsibility towards colleagues, towards the scientific community who wants to build on one’s results, and as a responsibility towards one’s own reputation and career.

In many quotes like this, being diligent is framed as a precondition for being able to assume other responsibilities (from publication to funding and ultimately the societal uptake of knowledge). It is further interesting to notice that in many instances, diligence is also framed as the only societal responsibility that a researcher can genuinely assume. Making diligence the core/sole responsibility may either be interpreted as not knowing how to assume (or not being educated about assuming) other responsibilities or as an implicit delegation of other responsibilities elsewhere.

Interestingly, what precisely being a responsible and diligent researcher entailed in this narrative was not always clear in the discussions. It is interesting to note that despite the rising importance of formalised codes of conduct at universities and other research institutes, researchers did not refer to any of these formal rules. Rather, diligence was often depicted as an embodied skill that is acquired over time and that, at some point, becomes a tacit part of research practices. As a researcher puts it, “*I just assume that … my attitude is right in that regard*” (PI11, German original quote). It is, however, also specific for this narrative that researchers talk a lot about specific practices of cultivating this tacit skill. Across career stages, exchange with colleagues was named as crucial to upholding high scientific standards, because data analysis will always leave grey zones of interpretation, as this principal investigator describes:

PI8: There is always a grey zone in which, … as a PhD student, you don’t know exactly. Or even as a group leader you have to speak about your research, what is significant and what not? And that’s when I consult my colleagues and have a look at what the state of current research is, what is published, what do they show … If you have doubts, it is important to talk about it … there are a lot of conversations at the institute and in the lab.[German original quote].

Maintaining a discourse about diligent practices and research integrity was seen as central to every research group, and particularly as a responsibility of the group leader rather than of the institution. Discussants referred to a range of different practices in which they saw this form of responsibility being trained and discussed.

In particular, principal investigators often expressed a concern about how to familiarize their students and staff with the tacit standards of good scientific practice in their specific labs. They also talked about concrete practices of creating such a culture, such as “*having very open discussions about every experiment*” (PI11, German original quote) and discussing the interpretation of raw data in lab meetings and journal clubs. Group leaders thus expressed a responsibility for creating a culture of transparency and for serving as role model for good scientific practice in these semi-institutionalised ways, as the following quote may exemplify:

PI8: I need to trust my people that they… show me the actual data. And to do that, I need to initially invest and explain: ‘Okay, this is right and this is wrong. Let’s look at this together or interpret this together…’ But you can never… be absolutely sure, this is a matter of trust! …ultimately, the boss is responsible, because… either you have a culture of transparency or not.[German original quote].

#### Communicating with Society

A second, though less frequent, narrative defining the meaning of responsibility in life science research related the responsibility of researchers for the practice of communicating with society. The following exchange between students expresses the high degree to which this concern is shared within the life science community:

LM1: [V]accination and GMOs [genetically modified organisms] … you can prove things and try to communicate things … but some people just will not accept … have we somehow failed as researchers … to assure people?

PhD3: That’s a good point …

PhD4: … [E]ducation of society is … one of the important responsibilities or for me as a scientist. … it is also my personal responsibility that people [are] not having … ideas of [the] Middle Ages … but of [the] twenty-first century.

In this narrative of responsibility, the scientists’ mission is described as to teach and convince the public, and to make people aware of the knowledge science produces. “The public” implied here often remains relatively unspecified, in the sense that there are no concrete societal actors or groups mentioned. Terms like “assuring” or “educating” further communicate that researchers see their responsibility in a one-way communication from scientists to “the public”. Communication from the public to scientists was not seen as a crucial part of this practice by most discussants. Rather, researchers tended to see their societal responsibility in raising scientific literacy. As in the quote above, communication is often framed as being particularly important in relation to the concern that scientific knowledge is not adequately considered in important societal decisions, and in the context of contemporary knowledge societies. Consider the following quote:

MS3: I chose the [card] “Public Intellectual”, because … especially communicating … things … to the public, adds this responsible dimension to a researcher; especially like nowadays where, like, so much knowledge is generated; but it fails to be communicated to the mass. So, I think that’s something that might make a good researcher into a responsible researcher.

In such narrative contexts, researchers explain that they want to prevent public decisions being made out of “fear” or based on “gut feeling” and want to contribute to making “educated decisions” (PI8, German original quote) by communicating science better. As the last sentence in the above quote conveys, communication is not seen as a core attribute of good researchers but rather as a non-mandatory add-on responsibility that at least some good researchers can also do without.

It is an interesting feature of this narrative that communication is usually not constructed as the individual responsibility of every researcher, but rather as a collective responsibility that the scientific community should recognise and assume:

PI8: I think this was maybe better in the past, that we had excited the society somehow. We had stars, our icons … Einstein … who had ‘rock star’-status … I think such people are extremely important … in research, to look and say: here is someone who represents this … metier, this group of people and it is important that we invest in that … And that can only develop if we learn, as researchers, to approach people who are not experts.[German original quote].

As in this quote’s mention of society’s realization that “it is important that we invest”, researchers tended to point to the scientific community’s failure to communicate well enough to convince societal actors to adequately support basic science with financial means. Communication as a practice thus appears as both a responsibility to society and a responsibility towards science itself.

A much rarer version of the communication-as-responsible-practice narrative includes interaction and mutual knowledge flows between science and society. As the following exchange shows, this version of the narrative also frames extra-scientific perspectives as being relevant for achieving responsibility in research practices:

MS7: [T]here are only so many things that you yourself could think of … Then you spread the knowledge, then you get other people thinking about it, and this is where you can get other ideas.

Overall though, communication often remains a rather abstract practice: even linear communication practices often remained unspecific, with few references to examples of when researchers had practised it. Even more so, interactive communication models were talked about in abstract ways, without mentioning specific practices of interaction or specific societal actors they might interact with.

#### Articulating Societal Relevance in Research Practice

The third, and most controversially discussed narrative was the active articulation of societal relevance. This articulation could be achieved either by being a researcher who is motivated by societal concerns and problems and who also considers this motivation in one’s choice of research topics and approaches, or by actively articulating the relevance of one’s research in direct interaction with societal actors (in the sense of entering into a dialogue about which knowledge would be relevant for actors outside science). The latter was marginal in the discussions, however, and was limited to specific institutional contexts of translational medicine.

Across the majority of our empirical material, however, this kind of articulation was seen as unusual for scientists, and often implicitly even in tension with notions of being a good basic researcher. Consider the following exchange:

PI10: [G]ood basic scientists don’t talk about relevance to others, they just want to know … they are interested by it and they’re excited by it …

I: Mhm, but the fact that it’s relatively easy in your case to argue relevance was not a reason for you to go in that direction?

PI10: No, of course not! No, absolutely not! I mean, you’re excited by your question and that’s all you’re interested in, really! Of course, you’re … hoping that others will be interested so they would hire you and give you a grant … so you do have to learn how to write the thing, but at the end, you’re writing to a scientist, you’re not writing to a non-scientist, right? So, the language is for insiders, let’s put it that way.

As in this quote, not engaging with questions of societal relevance is often attributed to “good basic scientists”, implicitly defining a motivation by societal issues as something that is not relevant to researchers. Even if societal relevance is used as an argument in grant writing, it is described as defining neither the research design nor a researcher’s personal identity:

PI8: Yes, this is an opportunity … doing cancer research is relatively easy [to argue to get funding]. Because I am in a First-World Country in which cancer is a problem and it is relatively easy to illustrate this as an important justification … for why we need to do this kind of research. But actually, it is not the primary reason. I do not consider myself a cancer researcher … Primarily, I am interested in the mechanism, how it works.[German original quote].

One important discursive reason for favouring inner-scientific curiosity over societal relevance as the driving force of researchers’ actions relates to an implicit concern for maintaining the autonomy of science. On a systemic level, virtually all discussants agreed that it should be first and foremost inner-scientific logics that decide which research directions are pursued, rather than the concerns of societal actors or their rationales. Consider this statement by a PI:

PI4: Research works if we all like it, if we think it’s useful, yeah. Certainly, we all do things that we believe … [are] useful, yeah, and then we are maybe good, but not if the others tell us what to do … I was very attracted by that sentence … ‘researchers should only follow the scientific curiosity’. Because I really think that’s the way that we do good science, yeah, relevant science …

Quite similarly to the previously cited PI, he starts with this strong statement equating inherent motivation with relevance and good science. Interestingly though, later in the discussion he explains how he is personally very interested in contributing to solving global problems, but that he does not see this motivation as a prerequisite for all researchers:

PI4: [T]he question [of global food security] … really interest[s] me personally. It motivates part of my research … but it’s not for everybody.

With this last segment of the sentence (“it’s not for everybody”), he places the articulation of societal relevance outside of what he considers to be the core responsibilities of every researcher. In fact, the view is widely shared in our material that societal concerns are—and should be—a personal matter. A master’s student for example picks up on what the above PI said by explaining that “*it’s more an individual trait*”, “*more related to … that researcher as a person*” (MS3). In that sense, articulating societal responsibility was seen as a responsibility that is not mandatory: it may be exercised by some researchers based on their personal conviction, but must not interfere with the pure curiosity (untouched by societal values) that should remain the main driving force of basic science.

Beyond the general form of this narrative, we observed two interesting trends: first, that younger researchers (mostly master’s students and to some extent PhD students who had been exposed to institutional conditions only for a limited time) found the idea of a closer articulation between societal issues and research directions to be much more intuitive and agreeable than the other participants did. And second, that researchers from different institutions had slightly different opinions on this issue: Researchers from a basic research institution that also promotes links to clinical research and whose leadership also emphasises social responsibilities (e.g. in hiring procedures) agreed more with the notion that research practices should be responsive to societal issues and concerns than researchers from a university institute. Even though these trends hint at the potential influences of institutional conditions on how researchers frame societal responsibilities, in our material these differences were merely differences in degree, and hence should be interpreted with caution.

Only a few researchers saw articulating societal relevance as a general responsibility of all publicly funded researchers. The following quote, in which a researcher frames societal relevance as an important sensitivity within basic research practices, thus represents a minority view:

PI12: We are responsible, because in a way we are funded by the public, right? So, the public somehow cares about what we do and cares to basically give some of their earnings to us – which I think is very important for us always to remember … So, this means that the work that we do should at the end… somehow benefit to the betterment of the society. This may not be very short-term, … But I hope that at some point our findings will kind of galvanize into something tangible for the society, so we should not basically lose sight of this and if there are ways that we could help, for example engage with the community, engage with the public, etc., this we should not ignore and we should actively seek for this kind of opportunities.

Again, with very few exceptions in the translational institutional contexts mentioned above, this narrative of responsibility was discussed purely in the abstract, without any references to concrete practices where this responsibility would be realized.

### Two Narratives that Stabilise the Broader Narrative Infrastructure for Being a Responsible Researcher

To understand what stabilises these three narratives within a broader narrative infrastructure related to societal responsibilities in research, it is instructive to look at what they share as common basic assumptions: the prevalence of a linear model of science-society relationships, on the one hand, and the notion that “not assuming societal responsibilities” is a collateral effect of institutional conditions, on the other. In the following, we will discuss both in turn.

#### A Linear Model of Science–Society Relations

Analysing the narratives identified in the previous section in-depth, it is possible to identify three shared implicit assumptions in the respective dominant positions about how the science-society relationship works best: (1) science is most valuable when undisturbed by societal logics or influences, (2) this autonomy of researchers guarantees that science will have a positive impact on society by providing new knowledge and (3) the societal relevance of knowledge will be discovered and taken up later for potential applications, by actors outside science. This imagination also tacitly implies a temporal order: that societal relevance is only recognisable after research findings have been published. The following exchange between three early stage researchers shows how smoothly researchers usually agree on this linear model when it comes to the societal implications of research.

PhD11: [T]his broader basic science is important, even if it doesn’t have some concrete goal … when you talk about CRISPR (a method allowing for gene editing) … that wasn’t an intentional thing, it just came out from other research. So, it’s basic research that then can be applied later … I think it’s only good for humanity just to do something because it’s interesting and to expand our knowledge of how the world works and what our place is … you get some … sort of, like a philosophical benefit.

PhD3: If you are not in building a knowledge base, you can’t improve society, can you? …

PD3: [I]n most cases you don’t know what you will get in the end, right? … I think as long as it is a contribution to the knowledge base – meaning it is something new and it extends current knowledge – … it can still be very valuable for the community or for society in the end … it is … already worth the effort.

Historically, it is of course possible to trace this linear model of innovation back to the post-World War II era in which Vannevar Bush in the US promoted the idea that basic science provides “scientific capital” and thus the core resource for technological progress (Bush [1945]1995). As Benoit Godin has shown, this linear model has since been stabilised by scientific institutions, such as statistical frameworks for measuring research and innovation performance (Godin 2006). Given this background, it is thus not surprising to be able to trace this linear model in the narratives of researchers. However, particularly within the past two decades, and building on a growing body of studies of innovation processes, this model has increasingly come to be questioned and replaced by more complex and interactive innovation models, such as those represented by the Responsible Research and Innovation framework. The stability and ongoing prevalence of the linear model in researchers’ narratives thus is remarkable.

For our purposes here, it is most interesting to consider what this linear model narratively does for researchers with regard to sketching a geography of societal responsibilities and to situating themselves within it. The above quotes show two arguments which are crucial for assuming and ascribing the societal responsibilities of scientists in this model. First, the application of knowledge and hence the responsibility for potential consequences of the knowledge produced is displaced both in time (later) and in relation to the actors applying it (not basic scientists). Second, (new) knowledge is seen as a cultural good, which in every instance has value for society. Taken together, these arguments narratively separate the realm of knowledge production from the settings in which societal relevance becomes visible and potential applications are explored. The implicit message of this linear model is that there is no value or benefit to be gained from considering societal relevance in what gets labelled as basic science. Much to the contrary, science works best if it is separated from such considerations. In wanting to position themselves as good researchers, what appears most intuitive for researchers who follow this linear rationale is to keep their distance from giving too much consideration to societal issues, expectations and concerns.

The displacement inherent in the linearity of the model was also invoked in the discussions to argue why scientists could not be responsible for the societal implications of their work. Consider this quote by a PI:

PI10: Regarding society, we are relatively safe … It is often so difficult to anticipate what the consequences will be … So, we have no reason to think about this …

However, it was precisely this question that was also often discussed controversially, particularly by younger researchers.^4^ This may be exemplified by the following debate:

PhD10: Every research can benefit society in the long run.

MS6: No, I don’t agree with that! Like, there is a lot of research done like from military, or they are making nuclear bombs, toxic and, so. … it’s not always as good. We should not have this biased point of view that science is the best thing in the world.

PD9: I was just thinking … Marie Curie discovered radioactivity, you can make out of it weapons, or … x-ray people which were wounded. … and the more scientists [are] top people, the more they can also show that there is a good side …

PD8: Yeah, science is neutral … but politics is different … how you apply is politics, but not science …

MS6: You cannot say [that]! No, it’s a responsibility! …these things are based on their inventions, and they are, like PD7 said, you should predict to some extent what … your discoveries can be applied for; so, it’s also partially your responsibility, you cannot just wash your hands…

Researchers who insisted that scientists have a responsibility to consider the later implications of their work for society remained in the minority in all discussions. A much stronger position in the debate was that scientists have a responsibility to defend the autonomy of science from efforts that would try to force research into directions, which are seen as focusing too strongly on relevance and application, and thus stifling scientific curiosity. Autonomy was then seen as the guarantee of societal benefit. Discussants saw a large risk in not conducting enough basic research and thereby breaking ground for future discoveries and societal improvements. In reference to CRISPR, a principal investigator, for example, speaks of the “*danger of not making discoveries*” when funds are directed too strongly to translational research and when researchers are not granted enough freedom to follow their interests. In such scenarios, basic science and potential opportunities are pictured as threatened by considerations of societal issues and concerns, as this quote by another PI shows:

PI6: I think … we are really at a point where there’s a strong threat that actually basic science is [not] going to be … possible to pursue. … when you look at, just to the European context … [if you are] just dedicated to basic science, it’s only ERC, that’s the only thing left. And it’s not even clear that beyond 2020 there will be funding of this program … EU hates that, political, politicians hate that, because that means they have, they have no control.

As in this quote, the linear model was dominant in all discussions we analysed, drawing a sharp distinction between science and society. Science is responsible for providing knowledge as a cultural good, but not for caring about its societal application and its consequences. Society is to support science unconditionally, and must not interfere with scientific curiosity in order not to endanger the proper functioning of science.

#### “Not Assuming Societal Responsibilities” as Collateral Effect of Institutional Conditions

Another cross-cutting feature of how researchers talk about societal responsibilities was the striking absence of reflections about the role of scientific institutions in promoting responsible research. It is interesting to observe that main scientific institutions (such as universities, research institutions or funding institutions) were hardly ever explicitly mentioned as facilitating concrete responsibility practices. Even in narratives related to research integrity such as “being a diligent researcher”, where institutional codes or regulations may be expected to play a crucial role, it was much rather the broader field-specific international scientific community that was seen as a key actor shaping responsibility practices.

The main scientific institutions on the other hand were instead described as curtailing scientists’ potential for acting responsibly through an excessive focus on competition and by an increasing short-term orientation:

PI8: [T]ime pressure, the weight of expectations—that prevents you from dealing with these things [i.e. societal responsibility], because in the end, I am not paid for it. Above all, I am paid for publishing … Everything else just takes away time… Initially, it is about having a job or having no job. … I cannot afford this luxurious question of whether this [my research] has some implications.[German original quote].

He refers in this quote to temporary employment arrangements that are often described as shifting attention to core responsibilities such as publishing. As in this quote, researchers talked about institutional conditions (“time pressure”, “weight of expectations”) as institutionalising the clear distinction between “their research” and “everything else”, which is framed as potentially taking time away from what researchers see as their core responsibility. In this sense, the way in which institutions measure performance (via publishing) and reward researchers is narrated here as stabilising the clear temporal and spatial gap between their actual practices and considerations of societal issues and concerns. Researchers described the way that institutions structure work environments and incentive structures as institutionalising the core values of the linear model of science-society relations. This often served as a justification for researchers to postpone concerns about the societal relevance of their research to an imagined future when they have achieved a permanent position. It also justified placing the consideration of societal issues and concerns outside the core requirements for making a career and – as in the quote above – allowed researchers to think of such considerations as a luxury.

Time pressure and evaluation structures were, for example, also referred to as factors that might potentially hamper researchers’ ability to assume responsibilities for diligently handling data:

PD3: I believe that the whole pressure that is built on the individual with regard to surviving within academia for example, getting funding and so on … is not (at) a good state at this point, because everything is basically pointing to a direction of producing clear results … black and white results and I think that’s most of the time not the way it is, but that’s obviously the only way to survive in this system. … I think the system needs to change in order to bring back diligence or a higher degree of diligence and also the possibility to be more honest.

As in the above quotes, the concrete role of institutions in creating conditions like “pressure” or a “weight of expectations” often remains quite vague. Or put differently, the actors who create these institutional conditions remain anonymous and are narratively hidden behind terms like “the system”. In this light, not assuming societal responsibilities appears as a collateral effect of how institutional conditions shape science. Linked to this, some researchers expressed a need to rebuild the whole research environment to better allow for responsible practices. One PI uses metaphors for the pace needed to succeed in contemporary academic career to argue for this:

PI12: [P]roperly or solidly demonstrating a finding will take [a] long time. You cannot do this in one, two, three years. So, what the, at the moment, the current system is always kind of, it is not a marathon, it is a sprint. You know, you run 100 m, you basically are almost like four hundred sprinters, you know. You kind of run 100 m, then you take the next step and then run. But for science, you need, you know, marathon runners, not sprinters. Because at the end, you are trying to discover something that hasn’t been shown or done before and this will not happen in a very short time. But currently, everything is based on very short time.

In summary, discussants were rather critical of scientific institutions’ ability to provide good conditions for responsible practices. The current speed of and competitive dynamics in scientific careers, for example, were often referred to as potentially compromising diligent research with a long-term perspective, or as hindering science communication and a reflection of societal relevance. At the same time though, discussants also seemed unable to concretely name which institutional actors could do things differently in order to better facilitate responsible research.

## Conclusions

The analyses of this paper show that researchers in the Austrian life sciences ascribe and assume responsibilities in reference to a strong culturally and institutionally anchored narrative infrastructure. In this concluding section, we show in three steps how this infrastructure pre-frames how researchers sketch what we call their geography of responsibilities. By sketching such a geography, researchers simultaneously assume certain responsibilities and delegate other responsibilities elsewhere.

As we have shown in this paper, the currently dominant narrative infrastructure tends to narrow down what researchers experience as their core responsibilities. This is a challenge particularly for widely discussed governance interventions, like Responsible Research and Innovation (RRI), that aim to change research practices to make them more responsive to societal issues and concerns. A rapidly growing body of work has proposed potential methods for realising this in practice (e.g. Felt et al. 2018b; te Kulve and Rip 2011), while a smaller but still important (e.g. Felt 2017; Lindner et al. 2016) corpus of literature has addressed the conditions under which these interventions can succeed or not in the current research system. This paper is mainly a contribution to the latter debate, and its main concluding argument is that the predominant culturally and institutionally anchored narrative infrastructure still does not fit with the core ideas of concepts like RRI.

### Responsibility Needs to Relate to Practices to Make Sense

A first important finding of this paper is that researchers were able to discuss and give meaning to narratives about responsibility if they were able to relate them to concrete moments in which this responsibility is practised by themselves or in their immediate social surroundings in science. This finding to a certain extent confirms Glerup and colleagues’ (Glerup et al. 2017) findings on how researchers frame their responsibilities: while scientific and political concepts of “responsibility” may be too abstract for researchers to relate to them, researchers can make sense of practices they know from their immediate surroundings. Most researchers, for example, could mention several ways in which they practiced being a diligent researcher (such as while discussing primary data in lab meetings, supervision meetings or in informal discussions about the interpretation of data within the lab or with colleagues). An interesting aspect of the diligence narration was, however, that researchers interpreted it as a responsibility towards their scientific community rather than as a responsibility towards society. On the other hand, when it came to communication with society or articulating societal relevance, practical examples were rare and limited to examples of classic science communication, such as giving interviews for newspapers or giving talks at schools. Even though in theory, some researchers were open to more interactive forms of communication, talking about it remained unspecific and without practical examples.

In terms of our geography metaphor, we could say that the narratives suggest that researchers’ concrete experiences about what diligence means in practice allowed them to place diligence very close to themselves within their geography of responsibilities, while practices of communication and societal relevance were rather situated elsewhere and were too far from them to matter for their research practices.

### The Dominance of the Linear Model Keeps Society at Arm’s Length

Linked to this, the empirical analysis has further shown that more complex and interactive models of science-society relationships (that inform concepts like RRI) have, so far, not gained acceptance in the life science community we observed. Rather, researchers drew on linear models of science-society relations as their primary narrative reference point in assuming and ascribing responsibilities. This linear model, as a crosscutting characteristic of their narrative infrastructure, leads researchers to sketch a geography of responsibility that keeps a temporal and spatial distance between them and broader societal responsibilities. This in turn narrows down the realm of what they feel responsible for: being a diligent researcher is defined as a primary responsibility, communicating to society is seen as an optional add-on, and articulating societal relevance is framed as the private business of those who feel the need to do so.

Even though this might seem paradoxical, it is the full embrace of some responsibilities that implicitly allows researchers to frame other responsibilities as irrelevant for how they plan and carry out their research. This clearly shows that, despite a range of policy initiatives such as RRI and other discursive shifts towards opening science up to society, the linear model still serves as cultural narrative structuring how basic researchers frame the societal responsibilities of science.

### Institutions Play a Key Role in Changing the Narratives of Responsibility that Researchers Live By

This paper argues that the persistence of the linear model of science-society relations cannot be explained by its discursive presence or its long history alone. The analysis of researchers’ narrations has also shown that the linear model is supported by the institutional structures they live in. One major finding of this paper is that while institutions are hardly ever directly mentioned in scientists’ discussions about societal responsibility, they are implicitly addressed as creating conditions that inhibit researchers’ capacities to experiment with and practice responsibility (e.g. interaction with societal actors). Universities’ and other research institutions’ value and incentive structures were seen as impelling researchers to be productive in terms of publications and the acquisition of research funding, i.e. two tasks that are strictly located on one end of the linear model. This served as a strong reference point for the argument that researchers’ primary responsibility lies in producing reproducible knowledge claims to build up an inner-scientific publication record. The title quote “I am primarily paid for publishing” epitomises this aspect of the narrative infrastructure, showing how strongly researchers’ priority-setting is also governed by how institutions value and evaluate different aspects of research practices and different kinds of responsibilities.

Two tacit trends within the broader narrative of articulating societal relevance also seem to confirm earlier findings (Fochler et al. 2016; Müller and de Rijcke 2017) that the duration of exposure to conditions within current research institutions (e.g. publication pressures) plays a role in shaping researchers’ attitudes. While master’s and PhD students still found it more intuitive to consider societal issues and concerns in their research practices, more senior researchers tended to place societal responsibilities further away from themselves. Also, institutional priorities in considering societal responsibility in governing research and hiring processes seemed to—at least to a degree—shape the way in which researchers framed their responsibilities. Both trends present valuable hypotheses for further research.

Our findings point to the crucial role of scientific institutions (universities, research institutions, professional associations or funding organisations) in creating new narratives, in establishing new reference practices and also in establishing new structural conditions that turn the consideration of societal responsibilities from a luxury into a standard procedure. In other words, implementing concepts like RRI also requires responsible scientific institutions that reflect on how the conditions they create shape opportunity structures for assuming societal responsibilities.

Hence, this paper concludes that RRI or related interventions cannot be successful by aiming to change the perspectives and practices of individual researchers alone. Institutions play a key role in at least two respects. First, meaningful interactions with society need to receive adequate attention and valuation within research institutions such as universities (e.g. in evaluation or hiring procedures). This requires institutions to engage in a reflection about the relative worth of such activities in relation to inner-scientific practices such as publication and grant acquisition (cf. Felt et al. 2018a). Concepts like RRI will not be able to achieve a better fit with researchers’ narrative infrastructures unless the connected practices become truly valued institutionally. Even though concepts such as RRI have gained ground in some funding structures (e.g. European framework programmes), these interventions are by definition temporally limited and may not be sustainably integrated into research practices and broader research cultures unless research institutions (like universities) develop more holistic plans for how to anchor societal responsibilities as a legitimate value in research practices.

Second, and partly following from this, institutions play a potential key role, not only in establishing time, space and resources for practising responsibility, but also in providing novel narratives that allow ideas related to RRI to enter researchers’ geographies in a way that matters for their practices. It is mostly at universities (and research institutions that provide PhD education) that researchers become socialised. Thus, universities are also key in creating time and space for meaningful responsibility practices in interaction with society (for examples, see the most recent debates in the *Journal of Responsible Innovation* or the collection of resources and inspiring practices by the RRI Tools project). This will allow researchers to experiment with and experience societal responsibilities as core responsibilities and articulate new meanings of responsibility in relation to their own practices. The effectiveness of such efforts will depend on considering existing narratives and reference practices within specific research cultures. This requires universities to work from the bottom up and to develop strategies from within research communities. Depending on the specifics of the respective research environments and research fields involved, local departments and professional associations or funding institutions also have a key role to play in fostering and adequately valuing such practices.

RRI, in whatever form, will only have a positive transformative effect on the current research system and its relation to society if it is successful in strongly altering the fabric of the core narratives researchers live by. This ties into earlier calls towards RRI scholarship to “develop a shared language of responsibility with scientists, and […] more actively address the political context of contemporary scientific research” (Glerup et al. 2017). However, the findings of this paper suggest that this will only be possible if core ideas embedded in the concept of RRI are embraced by institutions, not in the sense of paying lip service, but through actual change in what institutions do and what they value. On a policy level, this would require a shift from the notion of “removing barriers” for responsible behaviour towards “actively building” narrative and institutional infrastructures, i.e. the “responsibility conditions” (Felt 2017) that facilitate and reward responsible research in practice.

## Footnotes

1 https://www.rri-tools.eu/search-engine.

2 The cards used are published online and are accessible via this link: https://phaidra.univie.ac.at/view/o:690945.

3 Abbreviations: MS = master’s student, PhD = PhD student, PD = postdoc, PI = principal investigator, LM = lab manager, I = interviewer; most quotes are originally in English, other quotes are marked as originally in German.

4 It is interesting to note here that it was particularly young researchers (master’s and PhD students) who argued more radically for a better consideration of societal issues and concerns in research. This observation is in tune with the above-cited study by Fochler and colleagues (Fochler et al. 2016) showing that a longer socialization period within academia tends to narrow down evaluative registers to metrics.
